# What is the nature of peer interactions in children with language disorders? A qualitative study of parent and practitioner views

**DOI:** 10.1177/23969415211005307

**Published:** 2021-03-30

**Authors:** Vanessa Lloyd-Esenkaya, Claire L Forrest, Abbie Jordan, Ailsa J Russell, Michelle C St Clair

**Affiliations:** Department of Psychology, University of Bath, Bath, UK; Department of Psychology and Human Development, UCL Institute of Education, London, UK; Department of Psychology, University of Bath, Bath, UK

**Keywords:** Language disorder, developmental language disorder, specific language impairment, social skills, children

## Abstract

**Background and aims:**

Children with Language Disorders (LDs) can exhibit increased levels of social withdrawal, aggression and problems managing social conflicts. The reasons underlying this pattern of social interaction profiles remain unclear. This qualitative study aimed to document the nature of social interactions between children with LDs and their peers, and to evaluate explanations for their social behaviour, as understood by parents and practitioners.

**Methods:**

This study focused on children with LDs who spend school hours with other children with LDs. Three parent focus groups (n = 8) and three practitioner focus groups (n = 10) were conducted with parents of children aged 4–12 attending specialist language schools and practitioners working at these schools. This was a mixed clinical sample. All children of participating parents had LD as their primary area of need, which was the reason they required specialist schooling. Focus groups were conducted across two specialist schools in the UK between March and June 2018.

**Results:**

An inductive reflective thematic analysis of the data identified three themes; social knowledge, coping strategies, and emotional competence. Parents and school staff reported that children with LDs experience difficulties managing peer interactions due to a combination of challenges including difficulties with understanding and regulating emotions, and difficulties understanding social situations. Some of the children with LDs were described as having developed strategies to cope with their challenges, for example imposing structure on their social interactions to manage uncertainty, which has implications for their social interactions with peers.

**Conclusions:**

Children with LDs have difficulties understanding emotions, difficulties understanding their peer’s intentions and difficulties resolving conflict situations independently according to their parents and practitioners working with these children. Participants proposed a novel explanation that social withdrawal may be used adaptively by children with LDs to process information. This study demonstrates the complexity of the relationship between Language Disorders and peer interaction profiles.

**Implications:** Suggestions are offered regarding future research directions, such as investigating the specific contribution language skills make to children’s emotion understanding, to better understand the reasons for peer interaction difficulties in children with Language Disorders.

## Introduction

Communication difficulties among children are highly prevalent. A 2016 report using UK government census data found over 15% of children age 5–16 have Speech, Language and Communication Needs (SLCN) as their primary special educational need (Lindsay & Strand, 2016). Language Disorders are a type of communication disorder, which impact how people use and understand language. Language Disorder (LD) can be diagnosed in the absence of other known conditions which affect language development, in the case of Developmental Language Disorder (DLD), or it can be diagnosed in association with another neurodevelopmental condition, such as autism or a genetic syndrome (Bishop et al., 2016). Research shows individuals with LDs frequently experience higher than average levels of emotional, behavioural and social difficulties, including peer problems (Beitchman et al., 2001; Bercow, 2018; Bishop et al., 2016; Brownlie et al., 2016; Conti-Ramsden & Botting, 2008).

Social communication difficulties are one of the defining features of autism (Lord et al., 2020), often linked to “theory of mind” skills (Baron-Cohen et al., 1985; Leslie & Frith, 1988; Szumski et al., 2019). Although autistic children are typically reported to have lower functional social skills than children who have LDs (Barrett et al., 2004; Charman et al., 2015; Gibson et al., 2011; Loucas et al., 2008) and parents of autistic children are more concerned about their child’s peer relationships than parents of children with LDs (Lindsay et al., 2016), peer problems among children with LDs are nevertheless significantly elevated compared to “neurotypical” children (Bakopoulou & Dockrell, 2016; Conti-Ramsden & Botting, 2004; Levickis et al., 2017; Marton et al., 2005; Redmond & Rice, 2002). Furthermore, children with DLD are shown to be more socially isolated than “neurotypical” peers (Chen et al., 2018). Therefore, children with LDs are likely to experience social interaction challenges and may benefit from provision of services to support their social development (Leyfer et al., 2008; Toseeb et al., 2020).

Additionally, the language abilities of autistic children are highly variable and autism can co-occur with LD, where structural aspects of language are impaired (Bishop et al., 2016; Boucher, 2012; Tager‐Flusberg et al., 2005). While not discounting the inherent social differences associated with autism, such as reduced joint attention and reduced attention to other’s eyes and faces (Barrett et al., 2004; Hanley et al., 2014), researchers should not overlook the impact that having disordered language can have on children’s social interaction skills. Research focusing on the peer interaction experiences of a mixed clinical group of children with LDs is useful for identifying potential ways that language difficulties shape children’s socialising characteristics.

Theoretical approaches have been proposed to explain why children with LD experience challenges with peer relationships. For example, the Social Adaptation Model (Redmond & Rice, 1998) assumes that peers react to the limited verbal proficiencies of children with LD with certain biases, which can lead to high levels of peer rejection. Consequently, children with LD must find ways to cope with the demands of their social environment using their limited verbal abilities. Proposed coping strategies include initiating social interactions less frequently and relying more on adults for support (Redmond & Rice, 1998). Indeed, multiple studies find children with LDs have a tendency to engage in social withdrawal (Fujiki et al., 2001, 2014; Redmond & Rice, 1998, 2002). Social withdrawal is where a child isolates themselves from their peer groups (Rubin et al., 2009). A specific type of social withdrawal named reticence has been linked to LDs (Fujiki et al., 2004; Hart et al., 2004). This is where children observe their peers from a distance and are motivated to socialise, yet are reluctant to approach (Rubin et al., 2009). Social reticence is related to internalising difficulties (Graham & Coplan, 2012; Kiel et al., 2015; Sette et al., 2017) and research suggests that language disorder during childhood is a risk factor for social anxiety into adulthood (Brownlie et al., 2016). Therefore, the coping strategies that children with LDs use to cope with the social environment could be maladaptive to their social development (Redmond & Rice, 1998). An alternative view is that children with LD have a general underlying difficulty with working memory, which makes it harder for them to maintain social interactions with their peers, thus causing peer problems (Bishop, 2014a). Research suggests that the relationship between language and social development is complex; there is not necessarily a direct link between the severity of language problems and the severity of peer problems (Farmer, 2000) and the ability to engage in peer play relies on more than language skills alone (Guralnick et al., 2011; Roth & Clark, 1987).

Overall, children with LDs have an elevated risk of experiencing peer problems (Conti-Ramsden & Botting, 2004; Levickis et al., 2017; Lindsay & Dockrell, 2012). This is particularly concerning given that individuals with LDs have an increased risk of experiencing mental health difficulties during adolescence (Beitchman et al., 2001; Brownlie et al., 2016; Conti-Ramsden & Botting, 2008; Conti-Ramsden & Durkin, 2016; Durkin & Conti-Ramsden, 2010; Prizant et al., 1990; Strang et al., 2012; van Steensel et al., 2011) and research suggests that peer problems could mediate the relationship between LD and mental health difficulties (Forrest et al., 2018). Accumulating evidence supports the idea that social skills interventions, which focus on play and prosocial skills, might go some way to protect children with LDs from psychosocial difficulties (Toseeb et al., 2020; Toseeb & St Clair, 2020). Moreover, research suggests elevated emotional difficulties identified in children with LD could result from the interrelationship between early language difficulties and other developmental domains, including social interactions with peers (St Clair et al., 2019). It is therefore crucial that we develop a clear understanding of social development in children with LDs and explanations for their elevated peer problems. One way to achieve this is by conducting qualitative research.

Qualitative research which takes a phenomenological approach attempts to describe something happening by exploring the phenomena from the perspective of people who have experienced it themselves (Neubauer et al., 2019). There currently exists a dearth of qualitative research exploring the social development of children with LDs, as has been exemplified in a recent systematic review of studies researching the peer interaction skills of children with DLD (Lloyd-Esenkaya et al., 2020). It is important to listen to the views of the adults who have the most intimate knowledge of children with LDs as this can provide a real-life perspective on the social lives of these children. Unlike an interview between the researcher and participant, focus groups allow individuals to build on the ideas which arise from other individuals in the group. By listening to the comments made by other individuals, participants can reflect on their interpretation of their own views, allowing discussions to become deeper and more refined (Finch et al., 2014).

The study was conducted in specialist language schools. It should be noted that the current study was conducted in 2018, relatively soon after new recommendations for diagnosing DLD were made by an international consortium of experts (Bishop et al., 2016). In 2018 and even today, there is a lack of public awareness of DLD and this condition is frequently undiagnosed (Bishop, 2014b, 2017; Norbury et al., 2016; Thomas et al., 2019). The staff members working in these schools receive intensive training relating to LDs and are therefore well-placed to articulate their views on the underlying reasons for the social interaction characteristics of children with LDs. This is the first time a qualitative study specifically investigating the social skills of children with LDs attending specialist language schools has been conducted. This study aims to further our understanding of the nature of social interactions between children with LDs and their peers, as well as to evaluate different explanations for this behaviour as understood by parents and practitioners.

## Methods

### Design

A phenomenological approach was adopted in the current study to gain a detailed understanding of the social characteristics that parents and practitioners observe in children with LD, and an insight on the possible reasons for these characteristics. A topic guide providing open-ended questions was used within focus groups to allow participants to discuss their own observations of the children’s social behaviours and reflect on the experiences articulated by other parents or practitioners.

The topic guide (see Appendix 1) was used to lead discussions and the same order of questions was used each time. Following good practice for collecting qualitative spoken data (Braun & Clarke, 2013), focus group questions were open-ended and included prompts to encourage participants to provide additional detail about responses. Questions explored social interactions between children with LDs and their peers, focusing specifically on social withdrawal behaviour, challenging behaviour in social situations and children’s perceptions of peer relationships. Practitioners were asked about the nature of social interactions between children with LDs and their peers at school, who also had LDs. Parents were asked about the nature of their child’s social interactions with peers in general. Parents were free to discuss their child’s social interactions with peers from school, who also had LDs, or with peers outside of school, who might not have LDs.

The first and second author, who did not know any of the children discussed, facilitated the focus groups. Both had experience of researching the social and emotional development of young people with LDs and of working with children with and without language difficulties. It is important to recognise their previous experiences will have had an impact on the research by influencing the direction of conversation during some of the topics of discussion (Bourke, 2014).

### Recruitment and participants

Ethical approval was obtained from the Department of Psychology Research Ethics committee at the University of Bath (REF: 17-301). Pseudonyms are used throughout this paper to ensure the anonymity of the participants and children.

Focus groups were conducted across two specialist schools in the UK between March and June 2018. One was a specialist language day and residential school accepting primary school-aged children only. The second was a specialist language day and residential school accepting young people from primary school age through to college age. To be accepted into the schools, children needed to undergo an intensive multi-disciplinary assessment. Both schools provide an adapted curriculum to support children who have speech, language, and communication needs. Speech and Language Therapists (SLTs) work alongside teachers in both settings to tailor classes to the needs of their pupils. The same SLTs are involved in the admissions process when children are admitted to the schools.

We recruited practitioners and parents affiliated with specialist language schools to discuss their observations of children who have LDs, some associated with other conditions (e.g., autism or genetic conditions such as Fragile X Syndrome) while others would likely have met criteria for DLD. Practitioners working at the schools included Teachers, SLTs, and Occupational Therapists. Practitioners were invited to take part using posters placed on the staff notice boards.

The parents of children attending the schools were eligible to participate. The children were aged 4 to 12 years and had been diagnosed by a multidisciplinary team as having a Language Disorder as their primary area of need. Due to an especially wide catchment area for these specialist schools, it is common for the families to live far away. The lead contact for each school distributed information about the study to parents living within a half-hour radius of the school. Parents were spoken to at the school gates and given information sheets. Those who wanted to take part emailed the research team to register their willingness to participate.

Participants were eight parents and ten practitioners (Table 1). Two of the children of participating parents had LD associated with autism. It was not necessary to collect language assessment scores for the purpose of this study because we were satisfied that all children of the participating parents presented with a LD as their primary area of need. Separate focus group sessions were organised for parents and practitioners to encourage both participant groups to be as honest and forthcoming as possible in terms of the content and detail of their discussion. Additionally, implementation of parent-only sessions created homogeneity to encourage parents to exchange their experiences more willingly (Wibeck et al., 2007). It was assumed that they would draw on other parent’s views to talk about any similarities or differences they observed in their own children.

**Table 1.** Details of focus group participants and children of participating parents.

Focus group session	Participants who took part (pseudonyms)	Name of child (pseudonyms)	Gender of child	Diagnosis of language disorder associated with autism?	Age of children
1	Samantha, Parent	Lisa	Female	No	5 to 7
	Sienna, Parent	Megan	Female	No	
	Michelle, Parent	Kane	Male	Yes	
2	Rosemary, Practitioner				
	Johnny, Practitioner				
3	Jane, Parent	Amber	Female	No	8 to 12
	Mary, Parent	Neil	Male	No	
	Cassidy, Parent	Oscar	Male	Yes	
4	Sofia, Practitioner				
	Kate, Practitioner				
	Kassandra, Practitioner				
	Sasha, Practitioner				
5	Lesley, Parent	Paul	Male	No	6 to 9
	Thomas, Parent	Nicholas	Male	No	
6	Helena, Practitioner				
	Nathalie, Practitioner				
	Michaela, Practitioner				
	Paige, Practitioner				

### Procedure

All focus groups were conducted in private meeting rooms at the two schools. Refreshments were provided and all participants were reimbursed with a £10 book voucher. The first author provided an overview of the session at the start of each session. There was an opportunity to ask questions and written consent was obtained before audio recording began. The topics of discussion were displayed visually to guide participants through the structure of the session. Introductions to the topics were provided where appropriate to set the context for the coming questions; for example, giving an overview of research findings about patterns of social withdrawal in children with LDs. The duration of each focus group session was forty minutes to one hour. An audio recorder was used to record the sessions and the first author later transcribed these sessions verbatim.

### Analysis of data

Given the lack of any existing well-defined theory to explain the peer interaction behaviours of children with LDs, an inductive approach to analysis was adopted in this study via completion of inductive reflective thematic analysis (Braun & Clarke, 2006). A phenomenological epistemological approach was adopted in this study, assuming the perspective that an individual’s views of the world are subjective and it is possible to analyse their discussed experiences to find meaning, without using predefined theories (Braun & Clarke, 2016).

Thematic analysis is highly flexible (Braun & Clarke, 2013) and allows researchers to explore and examine themes across multiple data sets (Braun & Clarke, 2016). After transcribing each focus group session verbatim using Microsoft Word, the transcripts were imported into the qualitative data analysis software ATLAS.ti 8.7 (Muhr, 2019) to organise data and support the analyses of focus group transcripts. This made it possible for the first author to highlight ideas discussed by parents and practitioners relating to the children’s social interaction behaviours. ATLAS.ti 8.7 (Friese, 2014) allows researchers to label the transcripts with code names. By labelling similar ideas with the same code names it is possible to compare coded data across different transcripts. The first author used broad code names to begin with, such as “adult support” and “emotion”, and refined these to be more specific as they became more familiar with the nuances in the data.

Guidelines to conduct an inductive reflexive thematic analysis were followed in the current study (Braun & Clarke, 2006). The first author made initial notes regarding recurring ideas to become familiar with the data. The first author then independently generated initial codes across the data set and grouped these into initial themes which were discussed with the wider research team and revised and refined accordingly through multiple meetings. The codes were assigned names which all members of the team agreed on. Following these discussions, the first author was able to develop a thematic framework. All transcripts were then re-coded and further discussions took place. Mutual consensus across the research team was reached on the final codes and thematic framework.

### Steps to enhance qualitative research quality

After conducting the focus groups, both facilitators discussed their initial thoughts on the ideas expressed during the groups, any possible origins for these ideas and recorded such thoughts in a reflexive journal, thus enhancing the quality of the study by clearly situating the researchers within the context of their own research (Patnaik, 2013). Credibility checks were conducted in the form of multiple meetings with the wider research team to discuss the analyses over time (Elliott et al., 1999) and to ensure that themes were grounded in the data (Boije, 2010). Additionally, the research team ensured that a range of quotations across participant accounts were reported in the results section to ensure that voice was given to as many participants as possible (Lingard, 2019).

## Results

**Figure 1.**
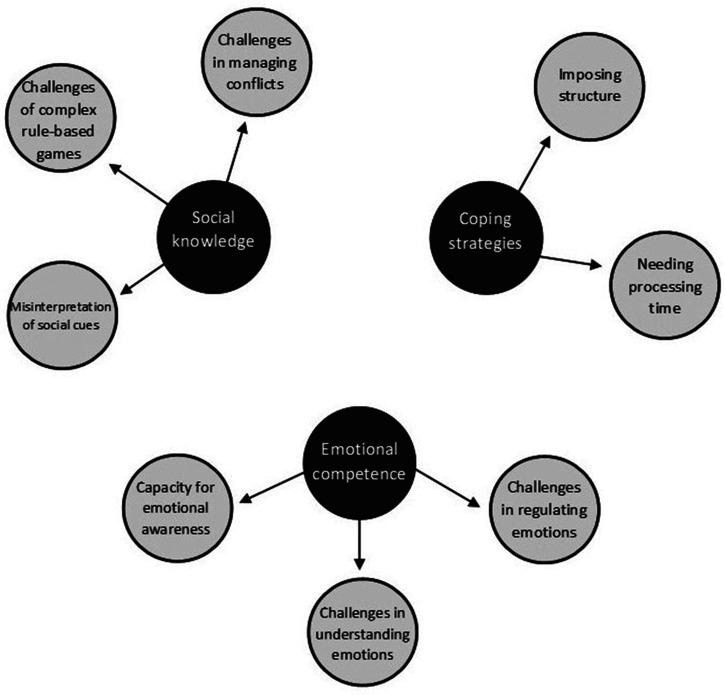
Thematic map to show themes and subthemes identified through thematic analysis as being important for explaining the social characteristics of children with Language Disorders.

An active period of engagement with the data resulted in the creation of three themes; social knowledge, coping strategies and emotional competence. Themes and subthemes are presented in Figure 1 and discussed in detail below, with quotations used to illustrate themes in greater detail.

### Social knowledge

Dominant throughout the accounts was a sense of the children with LD having a paucity of social knowledge around understanding their peers’ behaviours and managing social interactions with peers. The children were reported to experience challenges in accurately inferring the intentions of other children and at times mistakenly perceived peer behaviour as hostile. The children were also said to experience issues resolving peer conflicts, thought to result from their lack of understanding of the series of events preceding the conflict. The children engaged in social play, but this was confined to a small range of familiar games as they experienced difficulties mastering new games with complex rules. Within this theme, the subthemes “misinterpretation of social cues”, “challenges of managing conflicts”, and “challenges of complex rule-based games”, will be presented.

#### Misinterpretation of social cues

Children were reported to ascribe antagonistic intentions to peer behaviours. For example, a practitioner described how a child mistakenly bumping into another child may be interpreted as, “*“You’ve tripped me up”, well no, you both ran into each other*” (**Kassandra, Practitioner**). In this way, Kassandra recounts how it is common for a child in the specialist language school to wrongly assume that a peer would make a deliberate decision to behave in a malicious way towards them. The children with LD appear to possess a gap in their understanding of the motivations underlying their peer’s social behaviours and have a negative bias in their understanding of their peer’s behaviours, which is potentially damaging to their ability to form positive relationships with peers. If the children approach social situations with the misconception that their peers are being purposefully hostile, any opportunities to establish friendships with these peers could be overlooked.

Accurately perceiving their peer’s intentions is sometimes challenging for children with LDs; parents and practitioners explained that some of the children would use non-verbal, physical behaviours as an attempt to initiate play, to overcome their expressive language difficulties. When this happens, it is said the recipient child often perceives the behaviour to be unkind and is therefore likely to react in a negative way. This downward spiral of events is explained clearly by **Helena, Practitioner,** “*a lot of children who want to reach out to others, who tap other people on the arm, and it’s misconstrued as, “he hit me!”. Whereas actually it’s, “I actually want to make contact with you, and I want to play with you. But I haven’t got the mechanics that say, come and play, or chase me”, or whatever*”.

Parents and practitioners believe that the difficulties the children have in using language to express their intentions results in frequent misunderstandings. If the children do not know how to initiate interactions with their peers and rely on non-verbal behaviour to instigate play, their peers might mistakenly perceive their social behaviour as being aggressive and might therefore avoid further interactions with that child. Social knowledge is something which children with LDs require support to acquire.

#### Challenges in managing conflicts

Participants observed that children with LDs did not know how to resolve social conflicts. Conflicts were said to regularly occur because the children found it difficult to negotiate. As an example, **Jane, Parent** described the relationship between her daughter and another child who had LD as “*explosive*” because the children were unable to cooperate, as a result of the challenges associated with LDs. In reference to turn-taking, Jane said, “*oh no they don’t do that. Hence there’s a lot of falling out. No, no compromise whatsoever*”. Children with LD appear to have a paucity of knowledge around managing social situations in a way that avoids unnecessary conflicts. This could create problems for the children forming positive peer relationships because their peers might perceive them as being argumentative rather than prosocial and approachable, which might make them less well-liked among their classmates.

Participants also observed that the children with LDs did not understand the sequence of events leading to conflicts. This is clearly exemplified by **Johnny, Practitioner,** who described that: *“I quite often … get children come up to complain about somebody else’s, another child’s behaviour. But if you delve deeper into that, that other child could also have a grievance. And you don’t really know…where the issue has started. So, and they haven’t been able to tell you, and work out for themselves where it started…So you’re never sure, “now do I put this child on the bench? Do I put that child on the bench? Put them both on the bench?””.* Johnny explains how difficult it is for adults to support children with LDs to resolve situations of peer conflict. During the discussion, Johnny implied that the difficulties the children had in explaining the cause of a conflict extended beyond weak expressive language skills, saying, “*the actual processing of what’s going on I think can be really difficult for them*”, **Johnny, Practitioner**. Processing social situations appears to be very difficult for children with LD, suggesting that these children will likely benefit from the assistance of trained adults to learn effective strategies to resolve situations of peer conflict.

#### Challenges of complex rule-based games

Overall, the children with LDs were keen to socialise with their peers and enjoyed playing with and alongside others. However, the way in which they played was compromised by their language ability. The only group games the children were seen to participate in at school were those that were led by an adult and had highly familiar rules, such as football, or highly repetitive games with very simple rules. One practitioner, for example, spoke of a game they observed being played by the children with LD where one child is taken by their peer to “prison” and, “*once that prisoner gets caught,* “*oh, why don’t we take somebody else, and they’re going to prison”*’, **Rosemary, Practitioner**. Rosemary explained how the same “prison” game is played every day and the game is never extended to include new rules or to accommodate new characters in the game’s narrative. When asked whether her son participates in games, **Cassidy, Parent** says, “*Not as such…We’ve tried that with him but he’s- you know. Again, it’s the understanding, of rules and things*”. In this way Cassidy infers that her son has difficulty playing games with other people because he cannot master the rules needed to maintain engagement in the activity. **Nathalie, Practitioner** maintained that the games which groups of children with LD were able to participate in were simpler than those observed in groups of children with typical language development because, “*they haven’t got the language to elaborate on, and move the game forward*”. Without the ability to engage with others in games with complex rules, children with DLD may have reduced opportunities to interact socially with their peers. This could restrict their chances to learn important social skills such as negotiation and compromising.

### Coping strategies

While discussing the children’s social characteristics, participants described certain behaviours which could be attributed to coping strategies acquired as a response to language difficulties. The children with LD were said to demonstrate an overwhelming urge to impose structure on their social interactions and some perceived this as a way of enabling the children to cope with their anxiety surrounding social interactions. Other participants perceived social withdrawal as a coping strategy because this afforded children with LD the time for mental processing. An overview of the coping strategies are presented below through the subthemes “imposing structure” and “needing processing time”.

#### Imposing structure

Parents and practitioners described how children frequently made attempts to impose structure on the interactions they had with their peers at playtime. **Jane, Parent**, explains that when her daughter, with LD, engages in social interactions with peers, “*She needs to control that situation, and a lot of that will not necessarily be verbal, but physical as well”*. It is plausible that these efforts to impose a structure on their social interactions with peers enable the children to compensate for their lack of social knowledge. Participants considered that the children with LD were far more rigid in the ways they approached peer-play than children without a LD. One child was described as making efforts to control social situations by needing to be the winner in games, “*if someone else is playing cards with him, he has to win. He’s got to be in control of everything*” **Mary, Parent.** While this desire to be the winner in games is not uncommon in children, participants emphasised that the children with LD had a preoccupation with controlling peer interactions, and this was a central component of their social behaviour. For example, when asked what her child’s social interactions generally look like, **Sienna, Parent** explained that her daughter, “*likes to make sure that she’s in control, by saying certain repetitive phrases that go with set activities or times of day, or things that she’s doing*”. This desire to control social situations could have negative implications for peer interactions. If children use winning a game as a strategy to control peer interactions and perceive the goal to be winning, rather than companionship through sharing an activity with another person, they might fail to notice their peer’s attempts to forge friendships. This could leave peers with the impression that the child is disinterested in interacting with them socially and may reduce the likelihood that their peer will seek the child out as a play-partner in the future.

Some parents suggested that this need to control their social interactions came from the children’s desire to feel more secure, thus overcoming feelings of anxiety. For Jane’s daughter, Amber, this motivation to control social situations was closely tied to a sense of insecurity. **Jane, parent**, explains that social interactions are very difficult for Amber because “*she never knows quite what to say. Um, but* [she is] *also very controlling. Because she needs to be able to control that situation so she feels safe”* and how, “*there is like a default setting. And if she doesn’t know what to say, and doesn’t know what to do, she will automatically talk about Fireman Sam*”. Amber therefore manages these difficult social situations by changing the topic of conversation to one that she is highly familiar with, such as the television programme Fireman Sam. In this way, gaining control of social interactions occurs as a coping mechanism to manage the level of uncertainty associated with social situations. Anxiety during social situations was also described by **Thomas, parent,** who explains how his child will not join his peers in play “*until he feels it’s safe*”. Jane explained that her daughter becomes anxious in many situations, not merely peer interactions, when she does not know what will happen next and explained that for Amber, “*Everything has to be very, very predictable and planned in advance*”. It is possible that a similar process applies to the inflexible approach to play described in some of the children. It may be that children with LDs are able to engage in familiar games when their play partners play by their rules, but when the play interaction deviates from the child’s expectations, the child is unsure how to react, and the interaction is therefore vulnerable to falling apart. For some children with LD, imposing structure could become a way to overcome unpredictable social situations.

#### Needing processing time

Participants explained how some of the children with LDs preferred spending playtime alone to, “*process what’s happened during the morning*” (**Rosemary, Practitioner**). In this way, social withdrawal could be an adaptive strategy used by children with LDs to give themselves the opportunity to think about earlier events. A practitioner described the case of one child who, after spending time alone at playtime, then, “*comes back in, and then he’s ready to interact and learn*” (**Kassandra, Practitioner**). It seems that some children with LDs use playtime to process information to prepare for further episodes of learning and social interaction. Children with LDs might find lessons particularly challenging, due to their language content, and playtime could offer an opportunity to distance themselves from language processing. Under these circumstances, children with LDs might prefer to spend time alone, rather than actively engage themselves in social interactions which are accompanied by additional language demands.

### Emotional competence

Understanding and regulating emotions were reported by parents and practitioners as amongst the challenges faced by children with LDs when interacting with their peers. A detailed insight into the children’s emotional competencies as considered by participants will be presented here, through the subthemes “capacity for emotional awareness”, “challenges of understanding emotions”, and “challenges of regulating emotions”.

#### Capacity for emotional awareness

While discussing the children’s social development, participants discussed the strengths they recognised in the children’s emotional development. The children seem to have developed an awareness of other people’s emotions. This could lead to the children mirroring the same emotions. For example, **Johnny, Practitioner** explained that when one child in the class expresses a negative emotion, there can be a “*chain reaction*” around the class. Johnny elaborated that when one child in the class is feeling upset, the emotion can spread to other children in the class suddenly, resulting in a universal sense of feeling upset. A focus for these children appeared to be on noticing this emotion in their peers and reacting by displaying this same emotion themselves. It seems that children with LDs are sensitive to their peer’s emotional states and can react to these emotional states. In social situations, being sensitive to other’s emotions could have positive implications for a child’s peer relationships. If children are aware that another child is feeling upset, they may be able to manage their own behaviour to help the other child to feel better, which could improve the children’s affiliation with one another. The way that children reflect their peer’s emotions in their own could, however, be merely an automatic response, which does not involve conscious reasoning about their peer’s emotional states. It is therefore unclear whether the basic level of awareness of other’s emotional awareness shown by children with LD has any impact on their ability to manage their own social behaviours.

The children also appear to understand that other people’s emotions can result from their own behaviours. **Michelle, Parent** explained that her son “*doesn’t like it if anyone’s upset with him*” and goes on to explain that if he believes she is upset with him he might say, “*Please don’t be upset with me. I’m making you smile*”. This shows that children with LD are aware that their actions can change the emotions that other people feel, and these emotions can be expressed using words. If children are aware that their behaviour can make others experience different feelings, and these feelings can be labelled, they may be able to recognise when their behaviour has had a positive or negative impact on other’s emotions. This could help when they are learning how to interact with their peers in social situations. For example, by realising the effect that their behaviour has had on a peer’s feelings, they might learn to maximise the frequency of peer interactions which make others feel positive and minimise peer interactions which make others feel negative. In this way, being aware of other’s emotions could help children to manage their social behaviour.

#### Challenges in understanding emotions

While some of the children were able to give a brief description of their own emotions, there was variability, with some of the children finding it more difficult to describe their emotions. In reference to this, **Sienna, Parent** explains how her daughter has started a programme called the Zones of Regulation at school to learn about emotions, “*And they do the zones. And they just do um the blue zone, and the green zone. And so the happy, and the sad. And hitting makes people feel sad. So she’s, I think. She has to be taught, everything’s, she has to be trained: this results in this*”. Sienna highlights how her daughter needs to be taught about emotions in an explicit way as it is not something she has learnt on her own. Practitioners also commented that the children’s descriptions tended to be vague, and the challenges the children had in describing their feelings could interfere with their peer relationships. Practitioners believed that the children were unable to fully express their emotions to the level of detail expected of children their age because they only had a smaller vocabulary for emotion labels. For example, **Lesley, Parent** explains that when her child tries to express how he is feeling, “*very happy! Can also be happy. And a little happy? Also happy*”. Lesley’s child uses the same emotion term, happy, to describe how he is feeling, regardless of the level of intensity of his feelings. Being able to successfully express one’s feelings to others is an important skill for maintaining positive peer relationships.

If children are unable to give nuanced descriptions of their internal feelings it could be difficult for them to communicate to one another the motivations guiding their behaviour, and this could result in peer disputes. Indeed, **Cassidy, Parent** explained how her child regularly engages in conflict with a certain classmate because “*they can’t express to each other how they feel*”. Cassidy observed that the two children regularly upset one another because they were unable to talk about the impact of the other child’s behaviour on their emotions and now require constant adult supervision to keep them apart at playtime. Therefore, peer problems experienced by children with LDs might result from challenges in learning emotional vocabulary.

Participants also discussed how children with LDs appear to have difficulties accurately identifying their own emotions and the emotions felt by others, a key contributor to social competence (Denham et al., 2015). **Kate, Practitioner,** described a child who carried on doing something that was causing another child to become upset because she had not “*really equated, she’s [other child is] quite unhappy*”. Kate felt that the child failed to accurately identify the other child’s negative emotions, resulting in inappropriate behaviour that could be viewed as unsympathetic and insensitive. If the child’s peers perceive the child’s behaviour to be antisocial, they may form the impression that the child is unkind, and this could lower their opinion of the child. The emotion identification challenges observed in children with LD seem to extend to their own feelings. **Kassandra, Practitioner,** describes another child who, when asked how they feel, “*always says, “happy”. And she’s…had tears streaming down her face, she’s been really upset and she’ll go, “happy”*”. In this case the child is unable to match her feeling of sadness to the emotion label, “sad” to express how she feels. It is possible that poor receptive language skills limit children’s ability to accurately match vocabulary terms associated with emotions to the feelings felt by themselves and others. Without the language necessary to describe emotions, children with LD may not fully understand either their own or other children’s emotions. This would likely exacerbate the challenges children with LD have in explaining their feelings to their peers. Peer problems in children with LDs could therefore result from the children failing to accurately identify their own and their peer’s emotional states.

#### Challenges in regulating emotions

Participants observed that regulating emotions was an issue for the children with LDs. The children were described as having emotions which changed from “*nought to ninety*”, **Helena, Practitioner.** By this, Helena infers that the children move between different emotional states, which they experience very intensely, extremely rapidly. The children appear unable to control the speed at which they react emotionally to their environment. This could lead to difficulties when they are interacting with their peers. If a child’s emotions change at a faster rate than their peers are expecting, it will be difficult for their peers to predict the child’s imminent reactions to ongoing social events. These circumstances are described by one practitioner who expresses his surprise over the way that the children with LDs would react in a disproportionately aggressive manner towards their peers in response to small sources of provocation; “*Something will happen within…an interaction and all of a sudden,* “*I hit that person”…but it wasn’t for anything very big”,* **Johnny, Practitioner.** If a child becomes extremely upset very abruptly by something their peer has done, this could create social tensions because the child provides their peer with too little time to modify their behaviour, or explain their behaviour, in order to appease the child. The children with LDs appear to have difficulties independently regulating their emotions. **Sienna, Parent,** described how her child would fluctuate between extreme emotional states, “*and she can’t bring herself down, or lift herself out*”. By this, Sienna means that her child is unable to adopt strategies to calm herself down when she is feeling an extreme emotion. If children are unable to calm themselves down when they feel upset after being provoked by a peer in a social situation, it is likely to be difficult for them to manage the situation to create positive outcomes for their relationships with peers. Difficulties with emotion regulation could therefore be one factor contributing to peer problems in children with LDs.

## Discussion

This paper highlights that a combination of difficulties with language skills, understanding and regulating emotions, and understanding social situations seems to result in children with LD experiencing challenges with managing peer interactions. Some of the children with LD described in this study seemed to have developed coping strategies to deal with their challenges, including imposing structure on their social interactions and using playtime as an opportunity for processing previous events, which has implications for their social interactions with peers.

The schools in the current study were purposefully selected as it was felt the practitioners and parents, who were very familiar with the children’s language needs, would be able to talk at length, and with clarity, on the social behaviour of children with LD. As is the case with qualitative studies, the aim of this study is not to produce generalisable findings, but rather to gain an insight into the nature of social interactions between children with LDs and their peers from the perspective of the adults who observe them on a day-to-day basis. The following section will explain the findings in the context of how they contribute to our knowledge on the impacts of LDs on children’s social behaviour. It should be noted that the following section represents our interpretation of the data, but as with all qualitative research, alternative interpretations are entirely possible (Greenhalgh, 2016).

Parents and practitioners in this study agree that children with LD find social interactions challenging and tend to play alone or alongside their peers or engage in play with their peers that is low-level, involving only few rules. Although children with typical language development tend to only engage in games with simple rules between the ages of 5 to 7 (Johnson, 2015), participants in the current study implied that the games played by the children with LD were far simpler than one would expect for their age. Recognising that children with LD will find it hard to extend their playtime games to include more elaborate rules, and will find games with complex rules difficult to participate in is an important first step to supporting these children to actively participate in peer interactions. Children with LD will likely benefit from having the rules of games broken down and explained in multiple ways by someone with more advanced expressive language skills. Recent research suggests that play and prosocial behaviours may allow children with DLD to learn relationship skills, thus developing their social competence, which seems to protect against externalising problems during childhood (Toseeb et al., 2020). Therefore, additional playtime support for children with LD to understand the rules of playground games could increase opportunities to gain social skills that are learnt through play.

Some parents in this study suggested that their children used control as a coping strategy to overcome their feeling of uncertainty experienced during social interactions. An assertive style of peer-interaction has been found in other studies of primary school children with LDs (Fujiki & Brinton, 1991; Weitzner, 1981). Researchers have also suggested the rigid, inflexible interaction style that autistic children display during social interactions affords them the opportunity to retain control over unfolding social situations (Muskett et al., 2010). Whilst there is evidence suggesting children who are perceived as highly dominant perform poorly on sociometric scores of popularity (Parkhurst & Hopmeyer, 1998), practitioners should recognise that for children with LDs, behaving in a “controlling” manner could be a tool which helps them to participate in social situations. The fact that these children have the confidence to engage in peer interactions, even when they struggle to understand the language used around them, should be commended. Raising awareness of the underlying reason for unusually assertive styles of social interaction in children with LDs among teachers in mainstream may empower teachers to find ways of encouraging other children in the class to adapt to the social behaviour of children with LDs, to make it easier for these children to socialise.

When discussing the children’s social behaviours, participants explained how the children had difficulties accurately identifying their own and other’s emotions. A multitude of studies have found that autistic children often experience challenges in understanding other’s emotions (Harms et al., 2010; Neuhaus et al., 2019; Salomone et al., 2019). However, little is known about emotion understanding in children with LDs, outside of autism. Our finding that parents of children with LDs and their teaching staff believe these children to have relative weaknesses understanding emotions is therefore important because it suggests that emotional understanding difficulties might result from disordered language skills. Therefore, intervention tools which explicitly focus on emotion understanding might benefit multiple populations of children with LDs, not just autistic children. Furthermore, this finding supports a growing body of evidence showing children with DLD have difficulties recognising other’s emotions and inferring emotions from a situational context (Bakopoulou & Dockrell, 2016; Ford & Milosky, 2003; Griffiths et al., 2020; Merkenschlager et al., 2012; Spackman et al., 2006; Taylor et al., 2015; Vendeville et al., 2015). Future research could further explore the relationship between emotion understanding and social competence among children with LDs.

Participants explained that it was difficult for the children with LD to resolve conflicts independently. This supports previous evidence of weak conflict resolution skills and low levels of cooperative behaviour in children with LDs (Bakopoulou & Dockrell, 2016; Campbell & Skarakis-Doyle, 2011; Jahr et al., 2000; Liebal et al., 2008; Marton et al., 2005). This is concerning given evidence that children who are poor at reconciling conflicts with their peers, risk social rejection (Chung & Asher, 1996). It is unsurprising that peer conflict resolution would be harder for children with poor expressive language skills. To successfully repair conflict situations, children must negotiate, assert their position in a way which is not aggressive and should not surrender their own boundaries (Horowitz et al., 2008). The frequent peer conflicts children with LDs are observed to have are likely exacerbated if children misinterpret the intentions of their peers in the situation leading up to the conflict. Evidence of children with LDs misunderstanding of other’s intentions has been replicated in another qualitative study (Hambly, 2014). In this study the children with LD were suggested to have a negative bias, interpreting their peer’s behaviour as being hostile. This might prime the children to behave in a defensive way, which could lead to further hostilities and conflicts.

The issues described in the current study relating to peer conflicts could be exacerbated further still by the challenges the children were said to have in regulating their emotions. Higher expressive language skills in toddlers have been found to correlate positively with the ability to cope with frustration (Roben et al., 2013) and there is evidence to suggest that emotion regulation difficulties are detrimental to positive peer interactions (Denham et al., 2003). Possibly children with LDs are unable to use effective strategies that heavily rely on language, such as seeking more information from others or using self-distraction after provocation (Bendezú et al., 2018; Calkins & Hill, 2007; Kopp, 1989). Furthermore, they may have difficulties reframing their thoughts to self-regulate during upsetting situations and this might result in the frequent peer conflicts discussed in this study.

An important point discussed was that some of the children with LD avoided social interactions during playtime. At surface level, this is consistent with the findings generally reported that children with LDs are socially withdrawn (Brinton et al., 2000; Fujiki et al., 2001; Redmond & Rice, 2002). The withdrawal behaviour of children with DLD, in particular, tends to be interpreted as reticent behaviour (Fujiki et al., 2001). Reticence is associated with shyness and social phobia, and is thought to be maladaptive for children’s social interactions and relationships (Rubin et al., 2009). The current study offers an entirely new explanation. According to practitioners, playtime offers a window of opportunity for children with LDs to process their learning, because they can be alone, and thereby disengage from further language processing. In this way, social withdrawal can be seen to be an adaptive strategy for children with LDs to cope with school lessons. While it is necessary to consider the possibility that social withdrawal is used adaptively by some children with LDs, the consequences this might have on losing socialising opportunities should also be acknowledged. It may be necessary to provide children with LDs opportunities to socialise through group activities that are less reliant on verbal skills to allow them ample opportunity to engage in peer interactions. Another necessary step may be for teachers to shorten learning times for children with LDs and allow them a choice of relaxing activities to end the lesson. This might provide enough time for children with LDs to process their learning or abstain from further language processing, allowing them to take full advantage of their playtimes as a chance to socialise.

A feeling of uncertainty during social situations was said to be present in some of the children discussed, and this had an influence on their social interactions. A parent suggested that her child with LD felt insecure in social situations because she did not understand other’s behaviour and therefore could not predict what would come next. We might infer that the uncertainty associated with social situations made her daughter feel anxious. It is not unusual to find heightened levels of anxiety in individuals with different forms of LD (Beitchman et al., 2001; Brownlie et al., 2016; Kuusikko et al., 2008; Scott & Beidel, 2011; Spain et al., 2018; Voci et al., 2006). Understanding the manifestation of anxiety in this way is useful because it predicts that explicit teaching of social behaviours might help to reduce anxiety associated with social situations in children with LDs.

The current study makes an important contribution to the field by providing a detailed overview of the social characteristics of children with LDs and the possible reasons underlying these characteristics from the perspectives of the adults who have the most intimate knowledge of these children. By taking a qualitative approach, this paper provides a rich insight into the lived experiences of the “peer problems” found in primary school children with LDs. Children with LDs could have a paucity of social knowledge which makes it difficult for them to understand the intentions of their peers and resolve conflict situations and could result in highly assertive behaviour to gain control during interactions. The current study presents a novel finding that some children with LDs may use social withdrawal adaptively. This is a new perspective which has never been previously considered and warrants further investigation. Furthermore, this paper finds evidence that children with LDs have difficulties with emotion recognition and conflict management skills.

Nevertheless, this study cannot confirm whether children with LDs attending mainstream schools will show the same patterns of behaviour during peer interactions. The findings presented in this study reflect the experiences of children with LDs who spend all their school hours interacting with peers who also have LDs. Therefore, the findings are not necessarily representative of children with LDs attending mainstream schools who more frequently interact with peers who have typical language development. Peers in mainstream schools might use their more advanced language skills to scaffold play, repair relationships when conflicts arise, and to facilitate social interactions when there are misunderstandings. Therefore, a slightly different pattern of behaviours might be observed among children with LDs attending mainstream schools. Future studies should explore whether children with LDs attending mainstream schools experience the same difficulties with emotional competencies and understanding of social situations as the children discussed in this study.

Furthermore, the current findings are drawn from the observations of parents and school staff, who may have their own biases in how they interpret the children’s behaviour. The current study is somewhat limited because there is no inclusion of any self-report measure or direct observation of the children’s behaviour. Future qualitative studies could ask children with LDs about their own perceptions of their social behaviours and relationships, in combination with an observational tool, such as the Manchester Inventory for Playground Observation (MIPO) (Gibson et al., 2011).

Additionally, a heterogenous group of children with LDs are discussed in this study. It is entirely possible that a focus group study about children with a more specific LD diagnosis would draw different themes. The findings of this study should be used to influence the direction of future research on more precise forms of LDs that are so far under-researched with respect to social development.

The current study makes an important contribution to the field by providing a detailed overview of the social characteristics of children with LDs from the perspectives of parents and teaching staff. Further studies should now explore social knowledge, emotional competence and the coping strategies which influence social behaviours in more depth in more specific populations of children with LDs.

## Appendix 1. Topic guide for parent and practitioner focus group sessions.

**Table:**

Introduction	Facilitator welcomed all participants to the session and gave each individual a post-it note. Participants read information sheets and signed consent forms. Each person introduced themselves and added their name on a post-it note to a poster. Facilitator stated the ground rules for the session.
*Question for parents*	*Can you describe to me what your child’s social interactions generally look like?*
*Question for practitioners*	*Thinking about the children with language impairment, what do their social interactions generally look like?*
Prompts	How do they play?
	Can you give me some specific examples?
	How long do their games generally last?
Introduction to withdrawn style of behaviour	“We’re going to move on now to think about particular behaviours to do with joining in with others. A couple of studies looking at this have found that children who have impaired language skills are sometimes more likely to shy away from social interactions than children without impaired language. But this isn’t to say the same is true for everyone, and we still know very little about what might underlie this pattern of behaviour. We’re interested in hearing from you what your experience is of how your child interacts with other children. There are obviously no right or wrong answers here. We simply want to find out what your views are from your observations.”
*Question for parents*	*How does your child usually respond in situations where there are other children present?*
*Question for practitioners*	*What do you notice about withdrawal behaviour in children with language impairment?*
Prompts	Could you give me a good example?
	How does play usually begin when your child is with other children?
	Why do you think this is?
	Where does this behaviour come from?
*Question*	*At what age did the behaviours you are describing happen?*
Prompts	At what age did this behaviour appear?
	At what age did it disappear?
Introduction to challenging style of behaviour	“Now we’ll be thinking about behaviours relating to reactions to social situations. Another line of research has found a tentative link between impaired language skills and behaviours which could be considered challenging. Again, there is still a lot which is not understood here. It may be that this type of behaviour is more common in some children and less common in others, we simply don’t know yet. So the following questions are simply an opportunity for you to share your own observations if you feel comfortable to do so.”
*Question for parents*	*Can you tell me about any circumstances where your child perhaps behaved in a social context in a way which could be considered challenging?*
*Question for practitioners*	*What do you notice about challenging behaviour in children with language impairment?*
Prompts	If you can think of any examples, in what contexts have you seen this sort of behaviour happen?
	Why do you think this behaviour happens?
*Question for parents*	*Can you tell me about any times where your child has spoken to you about their relationships with their friends?*
*Question for practitioners*	*Can you tell me about any times these children have spoken to you about their relationships with their friends?*
Prompts	What do they say?
	Why do you think it is like this?
	How do you think they conceptualise their relationships with their friends?
*Question*	*Is there anything else that you think we should know that we haven’t yet covered?*
Prompts	Is there anything we have missed?
	Do you have any more comments to add?
